# An optimal epidural catheter placement site for post-cesarean section analgesia with double-space technique combined spinal–epidural anesthesia: a retrospective study

**DOI:** 10.1186/s40981-020-00405-9

**Published:** 2021-01-04

**Authors:** Yuya Murata, Kumiko Yamada, Yuto Hamaguchi, Soichiro Yamashita, Makoto Tanaka

**Affiliations:** 1 Department of Anesthesiology, University of Tsukuba Hospital, 2-1-1, Amakubo, Tuskuba, Ibaraki 305-8576 Japan; 2 Department of Anesthesiology, Institution of Medicine, University of Tsukuba, 1-1-1 Tennodai, Tsukuba, Ibaraki 305-8575 Japan

**Keywords:** Combined spinal–epidural anesthesia, Motor weakness, Numbness, Obstetric anesthesia, Patient-controlled epidural analgesia

## Abstract

**Background:**

Epidural anesthesia affects lower extremities, which often prevents early mobilization postoperatively. The incidence of numbness and motor weakness in the lower extremities with respect to epidural catheter placement site in cesarean section (CS) is uncertain. We aimed to investigate the effect of catheter placement site on postoperative lower extremities numbness and motor weakness in patients who received combined spinal–epidural anesthesia (CSEA) for CS including analgesic effects and optimal epidural placement site in CS.

**Methods:**

We retrospectively included 205 patients who underwent CS with CSEA at the University of Tsukuba Hospital between April 2018 and March 2020, and assessed numbness and motor weakness in the lower extremities. We also examined whether differences in the intervertebral space of epidural catheter placement and epidural effect on the lower extremities are related to analgesic effects. ANOVA and Mann–Whitney *U* test were used for statistical analysis.

**Results:**

The incidence of numbness and motor weakness were 67 (33%) and 28 (14%), respectively. All patients with motor weakness had numbness. A more caudal placement was associated with increased incidence of affected lower extremities. There was no significant difference in the analgesic effect depending on the catheter placement site. When the lower extremities were affected, the number of additional analgesics increased (*p* < 0.001). Patient-controlled epidural analgesia was used for fewer days in patients with motor weakness (*p* = 0.046).

**Conclusion:**

In CS, epidural catheter placement at T10–11 or T11–12 interspace is expected to reduce effect on the lower extremities and improve quality of postoperative analgesia.

## Background

Currently, combined spinal–epidural anesthesia (CSEA) for cesarean section (CS) is widely used in Japan. The combined use of epidural anesthesia (EA) allows both the intraoperative provision of additional anesthetic and postoperative analgesia. Thus, CSEA decreases the neuraxial block failure rate and may improve the quality of postoperative analgesia [1]. CSEA can be further divided into the single- and double-space technique. Most institutions in Japan, including our hospital, perform the double-space technique [2].

EA-associated neuropathies are very rare among patients without spinal cord diseases and with uneventful procedures [3, 4]. However, EA, when sited in lumber intervertebral space, may cause postoperative numbness and motor weakness in the lower extremities. These effects on the lower extremities can not only induce delayed early mobilization but also secondarily cause pressure sores [5] and common peroneal nerve palsy [6]. Therefore, there is a need for careful postoperative observation.

EA works at different spinal nerve levels depending on the catheter placement site [7]. To the best of our knowledge, there has been no study on the incidence of numbness and motor weakness in the lower extremities with respect to epidural catheter placement site in CS. In clinical practice, early mobilization after CS is very important for newborn care and prevention of venous thromboembolism (VTE). If EA with sufficient analgesic effect interferes with ambulation because of numbness and motor weakness in the lower extremities, the patient-controlled epidural analgesia (PCEA) must be at an insufficient setting for adequate analgesia or terminated too early. Therefore, the more caudal epidural is, the more likely to affect the lower extremities, and the analgesic effect is expected to be less.

This study investigated the incidence of numbness and motor weakness in the lower extremities depending on epidural catheter placement site after CS with double-space technique CSEA. Furthermore, we examined whether differences in the intervertebral space of epidural catheter placement and epidural effect on the lower extremities are related to analgesic effects. Finally, we determined the optimal epidural placement site for post-CS analgesia.

## Methods

This study was a single-center, retrospective study that was performed at the University of Tsukuba Hospital and approved by the University of Tsukuba Hospital Ethics Committee (R02-011). The ethics committee granted a waiver of written consent by opting out. This study was conducted in accordance with the current Declaration of Helsinki. We reviewed the medical records, including the anesthesia records, of consecutive patients who underwent CS with CSEA between April 2018 and March 2020. Patients with a body mass index > 28 were excluded since EA was not performed due to postoperative anticoagulation. In our hospital, the double-space CSEA technique was performed in all cases. The anesthetic procedure was performed in the right lateral decubitus position. The puncture site was determined through palpation. After epidural catheterization using a standard procedure, spinal anesthesia was performed with 0.5% hyperbaric bupivacaine 10–12 mg and fentanyl 10 mcg at the discretion of each anesthesiologist.

All the patients who received CSEA underwent PCEA with the CADD-Legacy ® PCA, Model 6300 (Smiths Medical MD, Minnesota, USA) device as postoperative analgesia starting from the end of surgery. The drug solution for PCEA was 0.2% ropivacaine and 3 mcg/mL fentanyl. The PCEA settings were determined for each case with basal infusion rate of 3–4 mL/h, a bolus of 2–3 mL, and a lockout of 10–20 min. Postoperatively, an acute pain service team changed the settings and terminated PCEA depending on the patient’s pain and adverse events. Other analgesics, such as diclofenac, celecoxib, acetaminophen, and pentazocine, were added as required by the patient. In case postoperative analgesia was insufficient, intravenous patient-controlled analgesia (IV-PCA) with fentanyl was applied.

Two physicians (MY and HY) determined the actual epidural catheter placement site from the postoperative radiograph that is routinely taken to confirm the absence of retained surgical items. The two physicians had not received special training in radiographic image interpretation. The effects on the lower extremities were not known before radiographic image interpretation. Differing opinions between the two physicians were resolved by discussion to a final decision. In case there was a gap between the actual and recorded placement sites, the vertebrae number between the actual and recorded site was recorded. The number was presented as a negative value in case the actual site was more caudal compared with the recorded site. In case the epidural catheter did not appear on radiograph, the recorded site was used as the actual placement site.

Data were collected until the third postoperative day and included patient characteristics; postoperative numbness, including discomfort and sensory loss upon touch; motor weakness (numbness or motor weakness of any degree was considered to be present if the patient complained or if there was a record of actual difficulty in walking due to weakness); the Prince Henry Pain Scale (PHPS) score (0 = no pain on coughing, 1 = pain on coughing but not on deep breathing, 2 = pain on deep breathing but not at rest, 3 = some pain at rest but desires no other analgesia, 4 = pain at rest, desires more analgesia) [8]; the number of additional analgesics, including IV-PCA; the period of PCEA use; actual epidural catheter placement site; and the difference in vertebrae number between the actual and recorded sites. When IV-PCA was used, the number of days used for it was counted as the number of additional analgesics.

Continuous variables were presented as mean (standard deviation). Ordinal variables and non-normally distributed data were presented as median [interquartile range]. The mean PHPS scores, number of additional analgesics, and period of PCEA use were compared between patients with and without postoperative numbness and motor weakness. All statistical analyses were performed using EZR (ver. 1.41). The Shapiro–Wilk test was used to determine the normality of the distribution. ANOVA and Mann–Whitney *U* test were used to determine the statistical significance of differences in all comparative data. Statistical significance was defined as *p* < 0.05.

## Results

Among 458 patients who underwent CS during the study period, 205 patients (45%) received CSEA. Table 1 shows the characteristics of these patients. The incidence of numbness and motor weakness due to postoperative EA were 67 (33%) and 28 (14%), respectively. All patients with motor weakness presented with numbness. There were 29 cases where the epidural catheter was out of the range that allowed for radiograph. A gap between the actual and recorded placement site was found in 98 patients (48%). Moreover, the average vertebrae number between the actual and recorded sites was − 0.3 (standard deviation = 0.9).

**Table 1:** Characteristics of patients who received cesarean section with CSEA

	*n* = 205
Age (years)	34 [30–38]
Height (cm)	157 [154–160]
Weight (kg)	60.2 [55.8–65.0]
Body mass index	24.4 [23.0–26.2]
Gestational period (weeks)	38 [37–38]
Emergency	84 (48%)
Postoperative numbness	67 (33%)
Postoperative motor weakness	28 (14%)
Mean PHPS value	
POD1	2.0 [1.5–2.7]
POD2	2.0 [1.0–2.0]
POD3	1.4 [1.0–2.0]
Period of PCEA use(days)	3 [3–4]
Number of additional analgesics up to POD 1-3	1 [0–2]
Actual placement of the epidural catheter	
T9–10	1 (0.5%)
T10–11	13 (6.3%)
T11–12	71 (34.6%)
T12–L1	85 (41.5%)
L1–2	33 (16.1%)
L2–3	2 (1.0%)
The gap between actual and recorded sites of the epidural catheter	− 0.3 (0.9)

Data are expressed as the number (%), mean (SD), or median [interquartile range]

*CSEA* combined spinal–epidural anesthesia, *PHPS* Prince Henry Pain Scale, *POD* postoperative day, *PCEA* patient-controlled epidural analgesia

**Fig. 1:**
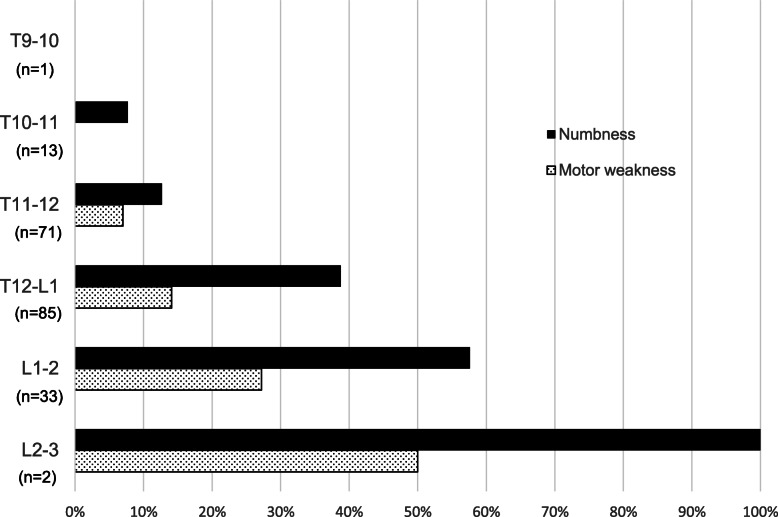
The incidence of lower extremities numbness and motor weakness with respect to epidural catheter placement site examined by radiograph

A more caudal placement was associated with increased incidence of affected lower extremities (Fig. 1). At T12–L1 interspace, the most commonly targeted site, the rate of numbness and motor weakness occurrence were 38.8% and 14.1%, respectively.

There was no significant difference in the analgesic effect based on the catheter placement site. There was no significant difference in the mean PHPS scores between patients with and without numbness and motor weakness (Tables 2 and 3); however, patients with numbness and motor weakness showed a significantly increased number of additional analgesics (*p* < 0.001). PCEA was used for fewer days in the motor weakness group (*p* = 0.046).

**Table 2:** Numbness in the lower extremities and analgesic effects

Numbness	Yes (*n* = 67)	No (*n* = 138)	*P* value^†^
Mean PHPS			
POD1	2.0 [1.3–2.7]	2.0 [1.5–2.7]	0.86
POD2	2.0 [1.0–2.5]	2.0 [1.0–2.0]	0.23
POD3	1.7 [1.0–2.0]	1.0 [1.0–2.0]	0.12
Number of additional analgesics up to POD1-3	2 [0–4]	1 [0–1]	< 0.001
Period of PCEA use (days)	3 [3–4]	3 [3–4]	0.14

Data are expressed as median [interquartile range]

*PHPS* Prince Henry Pain Scale, *POD* postoperative day, *PCEA* patient-controlled epidural analgesia

^†^Mann–Whitney *U* test

**Table 3:** Motor weakness in the lower extremities and analgesic effects

Motor weakness	Yes (*n* = 28)	No (*n* = 177)	*P* value^†^
Mean PHPS			
POD1	2.3 [1.7–3.0]	2.0 [1.3–2.7]	0.31
POD2	2.0 [1.2–2.4]	2.0 [1.0–3.0]	0.15
POD3	1.7 [1.0–2.0]	1.0 [1.0–2.0]	0.26
Number of additional analgesics up to POD1-3	3 [2–5]	1 [0–2]	< 0.001
Period of PCEA use (days)	3 [3–3]	3 [3–4]	0.046

Data are expressed as median [interquartile range]

*PHPS* Prince Henry Pain Scale, *POD* postoperative day, *PCEA* patient-controlled epidural analgesia

^†^Mann–Whitney *U* test

Other EA-associated adverse events included dural puncture in one patient and accidental catheter removal postoperatively in five patients. Two patients with suspected transient neurological symptoms (that improved after a few days) were found to have adverse events associated with the spinal anesthesia.

## Discussion

To the best of our knowledge, this is the first study to examine the relationship between epidural catheter location and epidural effects on the lower extremities in post-CS analgesia. The incidence of numbness and motor weakness was consistent with previous reports [9–12]; however, these previous studies were less accurate since ultrasound or radiography was not used to confirm the puncture and catheter placement sites. There was a higher incidence of numbness and motor weakness in patients with more caudal epidural catheter placement. Even minor differences in the intervertebral space of epidural catheter placement greatly affected the incidence of numbness or motor weakness in the lower extremities. This may be due to the spread of the drug solution in the epidural space. At the lower thoracic level, the drug solution tends to spread cephalad [7].

After CS was performed by CSEA, postoperative pain should be suppressed while minimizing the adverse EA effects on the lower extremities. In CS, epidural catheter placement in the lower thoracic vertebral level is recommended [10]. As indicated in the present study, epidural catheter placement at T12–L1 interspace is the most targeted in Japan for double-space technique CSEA [2]. Although there was no difference in the analgesic effect depending on the catheter placement site, we observed a high occurrence of numbness and motor weakness at the T12–L1 interspace. Further, compared with the double-space technique, the single-space technique greatly affects the lower extremities since an epidural catheter is usually placed at L3–4 interspace, which has lower patient satisfaction [13].

It is important to reduce numbness and motor weakness in the lower extremities to prevent secondary EA complications. Epidural effect on the lower extremities is a concern of VTE since it impedes early mobilization. In addition, a sensory block of the lower extremities can mask discomfort and pain, which leads to pressure sores and common peroneal nerve palsy caused by external pressure. A previous study reported nerve damage resulting from strangulation caused by elastic stockings for the prevention of VTE [14]. Fortunately, we did not observe any cases of pressure sores or neuropathy. Adverse effects on the lower extremities cannot be avoided with PCEA use. Detailed neurologic examination, including visual foot examination, as well as listening to the patient’s complaints, could be important postoperatively.

In the present study, patients with numbness and motor weakness in the lower extremities required additional analgesics and had early PCEA termination. Local anesthetic injection into the lumbar area might not enough provide analgesia in the lower thoracic dermatome. Similarly, PCEA might not be effectively used due to discomfort from numbness and mobilization difficulty. It is not clear from the present study which is the reason for lower effectiveness of the analgesia.

Not only should the target EA interspace be considered, but the potential deviation from the target interspace during puncture should also be considered. About half of our included patients had differences between the recorded and actual placement. Although Tuffier’s line is used as the landmark of the L4 vertebral body or the L4–5 interspace, the L3–4 interspace is often the landmark in pregnant women [15]. Furthermore, parturients might find it difficult to achieve the bending forward position for neuraxial anesthesia; moreover, it could be difficult to accurately identify the interspace by palpation alone. It appears that the target interspace should be decided based on the assumption that the actual puncture site is displaced from the target site by at most one interspace according to the standard deviation (Table 1).

Suprapubic transverse incision or lower abdominal midline incision is performed during CS. Providing analgesia in the T10–12 dermatome is required for postoperative pain relief. Our findings indicate that when performing CS anesthesia with double-space technique CSEA, the epidural catheter should be preferably placed at the T10–11 or T11–12 interspace. This could reduce the adverse effects on the lower extremities even with the displacement of the actual puncture site from the target site by one interspace, as well as effectively suppress the pain in the incision site.

Regarding postoperative analgesia, the PHPS score in the present study was approximately 2, a moderate score. In addition to the effective use of PCEA for post-CS analgesia, periodic NSAIDs and acetaminophen administration is a recommended multimodal method [16]. The effects of these analgesics on uterine contractions and breast-feeding should be fully discussed. However, there is a need to consider regular analgesics even when none are requested by the patient.

### Limitations

This study has several limitations. First, this retrospective study did not involve routine assessments of the actual numbness and motor weakness. Therefore, the incidence rate could have been underestimated. Second, there are two main types of post-CS pain: uterine contraction pain and wound pain, and PHPS could be inappropriate for evaluating post-CS pain. Third, since additional analgesics vary across cases, the same number of analgesics could have different analgesic effects and, therefore, affect the PHPS outcome. Fourth, since we could not detect epidural catheter placement in all postoperative radiographs, we presumed placement site in some cases. However, all epidural catheterization at the lumbar level were confirmed. A future prospective study that includes demographic-matched groups to eliminate confounding is needed to evaluate the incidence of EA adverse effects on the lower extremities and postoperative pain more accurately.

## Conclusion

In the present study, the post-CS incidence rates of numbness and motor weakness in the lower extremities were 33% and 14%, respectively. Epidural effects on the lower extremities were associated with the increased use of postoperative analgesics, which is indicative of insufficient pain control. In CS, epidural catheter placement at a more cephalad position, such as the T10–11 or T11–12 interspace, is expected to improve the quality of postoperative analgesia and reduce secondary complications of EA.

## Data Availability

The data that support the findings of this study are available from the corresponding author on reasonable reason.

## Abbreviations

CSEA: Combined spinal–epidural anesthesia
CS: Cesarean section
EA: Epidural anesthesia
VTE: Venous thromboembolism
PCEA: Patient-controlled epidural analgesia
IV-PCA: Intravenous patient-controlled analgesia
PHPS: Prince Henry Pain Scale
